# Supplementary material for: APOE Genotypes Modulate Inflammation Independently of Their Effect on Lipid Metabolism
Source: Int J Mol Sci. 2022 Oct 26;23(21):12947. doi: 10.3390/ijms232112947 (PMC9656824; doi:10.3390/ijms232112947)
Supplement: Supplementary file 1 [file ijms-23-12947-s001.zip › ijms-1931871-supplementary.pdf]

Supplemental Table S1. HDL composition and fractions according to *APOE* allele in a subsample from AWHS cohort

	**E2 allele[1] N= 132**	**E3 allele[2] N=813**	**E4 allele[3] N=183**	***p***
HDL Size (nm)	9.861 ± 0.211	9.860 ± 0.198	9.824 ± 0.165	0.151
Cholesterol in HDL (mmol/L)	1.244 ± 0.309	1.251 ± 0.273	1.203 ± 0.220	0.114
Triglycerides in HDL	0.162 ± 0.055	0.153 ± 0.047	0.155 ± 0.049	0.681
Small HDL particles (mol/l)	$4.709 \times 10^{-6} \pm 7.270 \times 10^{-7}$	$4.732 \times 10^{-6} \pm 5.314 \times 10^{-7}$	$4.714 \times 10^{-6} \pm 5.252 \times 10^{-7}$	0.395
Cholesterol in small HDL	0.426 ± 0.099	0.452 ± 0.079	0.453 ± 0.079	0.332
Triglycerides in small HDL	0.059 ± 0.020	0.057 ± 0.016	0.059 ± 0.017	0.961
Medium HDL particles (mol/l)	$1.804 \times 10^{-6} \pm 4.386 \times 10^{-7}$	$1.782 \times 10^{-6} \pm 3.652 \times 10^{-7}$	$1.726 \times 10^{-6} \pm 3.160 \times 10^{-7}$	0.067
Cholesterol in medium HDL	0.355 ± 0.103	0.351 ± 0.088	0.336 ± 0.079	0.056
Triglycerides in medium HDL	0.052 ± 0.017	0.050 ± 0.014	0.051 ± 0.015	0.763
Large HDL particles (mol/l)	$8.832 \times 10^{-7} \pm 4.754 \times 10^{-7}$	$8.813 \times 10^{-7} \pm 4.020 \times 10^{-7}$	$7.904 \times 10^{-7} \pm 3.173 \times 10^{-7}$	0.087
Cholesterol in large HDL	0.251 ± 0.155	0.246 ± 0.133	0.216 ± 0.102	0.047
Triglycerides in large HDL	0.028 ± 0.015	0.028 ± 0.013	0.027 ± 0.012	0.783
Very large HDL particles (mol/l)	$4.044 \times 10^{-7} \pm 1.553 \times 10^{-7}$	$4.009 \times 10^{-7} \pm 1.455 \times 10^{-7}$	$3.809 \times 10^{-7} \pm 1.218 \times 10^{-7}$	0.199
Cholesterol in very large HDL	0.204 ± 0.079	0.199 ± 0.072	0.194 ± 0.065	0.365
Triglycerides in very large HDL	0.017 ± 0.014	0.015 ± 0.011	0.016 ± 0.011	0.819

Quantitative variables were expressed as mean ± standard deviation. The *p* value was adjusted by age, sex BMI. [1]E2 allele included subjects with E2/2 and E2/3 *APOE* genotype. [2]E3 allele included only subjects with E3/3 *APOE* genotype. [3]E4 allele included subjects with E3/4 and E4/4 *APOE* genotype.

Supplemental Table S2. VLDL composition and fractions according to *APOE* allele in a subsample from AWHS cohort

	**E2 allele[1] N= 132**	**E3 allele[2] N=813**	**E4 allele[3] N=183**	***p***
VLDL Size (nm)	36.91 ± 1.599	36.62 ± 1.348	36.73 ± 1.337	0.416
Cholesterol in VLDL	0.836 ± 0.336	0.815 ± 0.290	0.863 ± 0.304	0.491
Triglycerides in VLDL	1.070 ± 0.736	0.953 ± 0.609	1.023 ± 0.655	0.674
Very small VLDL particle (mol/l)	4.333×10^{-8}± 1.028×10^{-8}	4.459×10^{-8} ± 1.016×10^{-8}	4.610×10^{-8} ± 9.807×10^{-9}	0.101
Cholesterol in very small VLDL	0.266 ± 0.064	0.277 ± 0.064	0.287 ± 0.062	0.040
Triglycerides in very small VLDL	0.123 ± 0.042	0.119 ± 0.036	0.123 ± 0.037	0.860
Small VLDL particle (mol/l)	3.540×10^{-8} ± 1.405×10^{-8}	3.384×10^{-8} ± 1.189×10^{-8}	3.557×10^{-8} ± 1.217×10^{-8}	0.863
Cholesterol in small VLDL	0.251 ± 0.082	0.253 ± 0.075	0.267 ± 0.073	0.201
Triglycerides in small VLDL	0.280 ± 0.139	0.258 ±0.115	0.272 ± 0.120	0.762
Medium VLDL particle (mol/l)	2.143×10^{-8} ± 1.396×10^{-8}	1.936×10^{-8} ± 1.164×10^{-8}	2.080×10^{-8} ± 1.251×10^{-8}	0.781
Cholesterol in medium VLDL	0.206 ± 0.119	0.191 ± 0.099	0.204 ± 0.106	0.964
Triglycerides in medium VLDL	0.368 ± 0.259	0.328 ± 0.216	0.353 ± 0.232	0.683
Large VLDL particle (mol/l)	6.149×10^{-9} ± 5.827×10^{-9}	5.213×10^{-9} ± 4.873×10^{-9}	5.734×10^{-9} ± 5.292×10^{-9}	0.624
Cholesterol in large VLDL	0.086 ± 0.079	0.073 ± 0.065	0.080 ± 0.070	0.684
Triglycerides in large VLDL	0.206 ± 0.199	0.174 ± 0.167	0.191 ± 0.181	0.595
Very large VLDL particle (mol/l)	9.738×10^{-10} ± 1.166×10^{-9}	7.738×10^{-10} ± 9.417×10^{-10}	8.793×10^{-10} ± 1.026×10^{-9}	0.613
Cholesterol in very large VLDL	0.019 ± 0.023	0.015 ± 0.018	0.017 ± 0.020	0.782
Triglycerides in very large VLDL	0.060 ± 0.071	0.047 ± 0.058	0.054 ± 0.063	0.570
Extremely large VLDL particle (mol/l)	2.062×10^{-10} ± 2.512×10^{-10}	1.607×10^{-10} ± 1.892×10^{-10}	1.841×10^{-10} ± 2.028×10^{-10}	0.647
Cholesterol in extremely large VLDL	0.007 ± 0.008	0.006 ± 0.007	0.007 ± 0.007	0.890
Triglycerides in extremely large VLDL	0.031 ± 0.038	0.024 ± 0.029	0.028 ± 0.031	0.622

Quantitative variables were expressed as mean ± standard deviation. The *p* value was adjusted by age, sex BMI. [1]E2 allele included subjects with E2/2 and E2/3 *APOE* genotype. [2]E3 allele included only subjects with E3/3 *APOE* genotype. [3]E4 allele included subjects with E3/4 and E4/4 *APOE* genotype

Supplemental Table S3. LDL composition and fractions according to *APOE* genotypes in a subsample from AWHS cohort

	**E2/E2 (N=3)**	**E2/E3 (N=129)**	**E3/E3 (N=813)**	**E3/E4 (N=171)**	**E4/E4 (N=12)**	***p***
**LDL size**	23.7 ± 0.10	23.6 ± 0.083	23.5 ± 0.074	23.5 ± 0.069	23.5 ± 0.080	0.322
**Cholesterol in LDL**	0.800± 0.131	1.566 ± 0.462	1.756 ± 0.486	1.859 ± 0.474	1.616 ± 0.419	**0.006**
**TG in LDL**	0.152 ± 0.028	0.186 ± 0.057	0.187 ± 0.053	0.191 ± 0.050	0.208 ± 0.056	0.304
**Small LDL particle**	$1.008 \times 10^{-7} \pm 1.531 \times 10^{-8}$	$1.650 \times 10^{-7} \pm 4.144 \times 10^{-8}$	$1.799 \times 10^{-7} \pm 4.365 \times 10^{-8}$	$1.891 \times 10^{-7} \pm 4.16 \times 10^{-8}$	$1.734 \times 10^{-7} \pm 3.567 \times 10^{-8}$	**0.009**
Cholesterol in small LDL	0.130 ± 0.032	0.283 ± 0.089	0.322 ± 0.092	0.341 ± 0.089	0.293 ± 0.083	**0.005**
TG in small VLDL	0.033 ± 0.009	0.033 ± 0.013	0.032 ± 0.011	0.033 ± 0.011	0.038 ± 0.013	0.388
**Medium LDL particle**	$7.992 \times 10^{-8} \pm 1.212 \times 10^{-8}$	$1.401 \times 10^{-7} \pm 3.755 \times 10^{-8}$	$1.545 \times 10^{-7} \pm 3.974 \times 10^{-8}$	$1.630 \times 10^{-7} \pm 3.811 \times 10^{-8}$	$1.473 \times 10^{-7} \pm 3.261 \times 10^{-8}$	**0.006**
Cholesterol in medium LDL	0.211 ± 0.046	0.462 ± 0.148	0.525 ± 0.154	0.559 ± 0.149	0.479 ± 0.133	**0.005**
TG in medium LDL	0.039 ± 0.006	0.050 ± 0.016	0.051 ± 0.145	0.052 ± 0.013	0.057 ± 0.015	0.133
**Large LDL particle**	$1.051 \times 10^{-7} \pm 1.209 \times 10^{-8}$	$1.740 \times 10^{-7} \pm 4.248 \times 10^{-8}$	$1.894 \times 10^{-7} \pm 4.576 \times 10^{-8}$	$1.984 \times 10^{-7} \pm 4.482 \times 10^{-8}$	$1.807 \times 10\text{-}^{7} \pm 3.871 \times 10^{-8}$	**0.012**
Cholesterol in large LDL	0.438 ± 0.056	0.819 ± 0.228	0.909 ± 0.242	0.959 ± 0.238	0.843 ± 0.205	**0.008**
TG in large LDL	0.082 ± 0.013	0.102 ± 0.030	0.103 ± 0.028	0.105 ± 0.026	0.113 ± 0.030	0.254

Quantitative variables are expressed as means ± standard deviations. The *p* value was adjusted by age, sex and BMI.

Supplemental Table S4. HDL composition and fractions according to *APOE* genotypes in a subsample from AWHS cohort

	E2/E2 (N=3)	E2/E3 (N=129)	E3/E3 (N=813)	E3/E4 (N=171)	E4/E4 (N=12)	*p*
**HDL size**	9.66±0.12	9.86±0.21	9.86± 0.20	9.83±0.17	9.80±0.16	0.183
**Cholesterol in HDL**	0.96±0.18	1.25±0.31	1.25±0.27	1.21±0.22	1.18±0.23	0.163
**TG in HDL**	0.19±0.05	0.16±0.06	0.15±0.05	0.15±0.05	0.18±0.07	0.754
**Small HDL particle**	$4.77x\ 10^{-6}$ $\pm 2.26 \times 10^{-7}$	4.71×10^{-6} $\pm 7.35 \times 10^{-7}$	4.73×10^{-6} $\pm 5. \times 10^{-7}$	4.703×10^{-6} $\pm 5.34 \times 10^{-7}$	4.90×10^{-6} $\pm 3.45 \times 10^{-7}$	0.539
Cholesterol in small HDL	0.35±0.07	0.43±0.099	0.45±0.08	0.45±0.08	0.44±0.09	0.317
TG in small HDL	0.07±0.02	0.06±0.02	0.06±0.016	0.06±0.016	0.07±0.02	0.936
**Medium HDL particle**	1.70×10^{-6} $\pm 8.92 \times 10^{-8}$	1.81×10^{-6} $\pm 4.43 \times 10^{-7}$	1.78×10^{-6} $\pm 3.65 \times 10^{-7}$	1.72×10^{-6} $\pm 3.20 \times 10^{-7}$	1.81×10^{-6} $\pm 2.48 \times 10^{-7}$	0.115
Cholesterol in medium HDL	0.31±0.04	0.36±0.10	0.35± 0.09	0.34±0.08	0.35±0.06	0.093
TG in medium HDL	0.06±0.01	0.05±0.02	0.049±0.01	0.05±0.01	0.06± 0.02	0.838
**Large HDL particle**	4.45×10^{-7} $\pm 3.85 \times 10^{-7}$	8.93×10^{-7} $\pm 4.74 \times 10^{-7}$	8.81×10^{-7} $\pm 4.02 \times 10^{-7}$	7.91×10^{-7} $\pm 3.18 \times 10^{-7}$	7.74×10^{-7} $\pm 3.16 \times 10^{-7}$	0.123
Cholesterol in large HDL	0.12±0.11	0.25±0.15	0.246±0.13	0.216±0.10	0.208±0.10	0.070
TG in large HDL	0.02±0.01	0.028±0.01	0.028±0.01	0.027±0.01	0.031±0.014	0.746
**Extra-large HDL particle**	2.58×10^{-7} $\pm 3.07 \times 10^{-8}$	4.08×10^{-7} $\pm 1.55 \times 10^{-7}$	4.001×10^{-7} $\pm 1.46 \times 10^{-7}$	3.83×10^{-7} $\pm 1.19 x10^{-7}$	3.49×10^{-7} $\pm 1.56 \times 10^{-7}$	0.191
Cholesterol in extra-large HDL	0.15 ± 0.01	0.21 ± 0.08	0.20 ± 0.07	0.20 ± 0.06	0.18 ± 0.08	0.331
TG in extra-large HDL	0.018 ± 0.01	0.017 ± 0.01	0.015 ± 0.01	0.016 ± 0.01	0.019 ± 0.015	0.805

Quantitative variables are expressed as means ± standard deviations. The *p* value was adjusted by age, sex and BMI.

Supplemental Table S5. VLDL composition and fractions according to *APOE* genotypes in a subsample from AWHS cohort

	E2/E2 (N=3)	E2/E3 (N=129)	E3/E3 (N=813)	E3/E4 (N=171)	E4/E4 (N=12)	*p*
**VLDL size**	38.608±1.196	36.871±1.589	36.618±1.348	36.693±1.293	37.275±1.851	0.443
**Extra-small VLDL particle**	$3.596 \times 10^{-8} \pm 4.782 \times 10^{-9}$	$4.351 \times 10^{-8} \pm 1.032 \times 10^{-8}$	$4.459 \times 10^{-8} \pm 1.016 \times 10^{-8}$	$4.613 \times 10^{-8} \pm 9.852 \times 10^{-9}$	$4.570 \times 10^{-8} \pm 9.543 \times 10^{-9}$	0.118
Cholesterol in extra-small VLDL	0.209 ± 0.0189	0.268 ± 0.064	0.277 ± 0.064	0.288 ± 0.062	0.2698 ± 0.059	0.059
TG in extra-small VLDL	0.126 ± 0.031	0.123 ± 0.042	0.119 ± 0.036	0.122 ± 0.036	0.138 ± 0.034	0.788
**Small VLDL particle**	$4.042 \times 10^{-8} \pm 1.262 \times 10^{-8}$	$3.529 \times 10^{-8} \pm 1.410 \times 10^{-8}$	$3.384 \times 10^{-8} \pm 1.189 \times 10^{-8}$	$3.530 \times 10^{-8} \pm 1.199 \times 10^{-8}$	$3.941 \times 10^{-8} \pm 1.464 \times 10^{-8}$	0.846
Cholesterol in small VLDL	0.2399 ± 0.055	0.251 ± 0.083	0.253 ± 0.075	0.266 ± 0.074	0.269 ± 0.074	0.237
TG in small VLDL	0.354 ± 0.134	0.278 ± 0.139	0.258 ± 0.115	0.269 ± 0.117	0.320 ± 0.152	0.812
**Medium VLDL particle**	$2.913 \times 10^{-8} \pm 1.475 \times 10^{-8}$	$2.126 \times 10^{-8} \pm 1.395 \times 10^{-8}$	$1.936 \times 10^{-8} \pm 1.165 \times 10^{-8}$	$2.046 \times 10^{-8} \pm 1.210 \times 10^{-8}$	$2.568 \times 10^{-8} \pm 1.728 \times 10^{-8}$	0.828
Cholesterol in medium VLDL	0.263 ± 0.121	0.205 ± 0.119	0.191 ± 0.099	0.202 ± 0.103	0.239 ± 0.143	0.942
TG in medium VLDL	0.519 ± 0.276	0.365 ± 0.258	0.328 ± 0.216	0.346 ± 0.224	0.448 ± 0.323	0.736
**Large VLDL particle**	$9.439 \times 10^{-9} \pm 6.063 \times 10^{-9}$	$6.073 \times 10^{-9} \pm 5.824 \times 10^{-9}$	$5.213 \times 10^{-9} \pm 4.873 \times 10^{-9}$	$5.567 \times 10^{-9} \pm 5.064 \times 10^{-9}$	$8.114 \times 10^{-9} \pm 7.772 \times 10^{-9}$	0.689
Cholesterol in large VLDL	0.132 ± 0.081	0.085 ± 0.079	0.073 ± 0.065	0.078 ± 0.067	0.111 ± 0.03	0.745
TG in large VLDL	0.318 ± 0.208	0.203 ± 0.199	0.174 ± 0.167	0.185 ± 0.173	0.273 ± 0.267	0.660
**Extra-large VLDL particle**	$1.555 \times 10^{-9} \pm 1.119 \times 10^{-9}$	$9.603 \times 10^{-10} \pm 1.167 \times 10^{-9}$	$7.738 \times 10^{-10} \pm 9.417 \times 10^{-10}$	$8.467 \times 10^{-10} \pm 9.783 \times 10^{-10}$	$1.344 \times 10^{-9} \pm 1.536 \times 10^{-9}$	0.690
Cholesterol in extra-large VLDL	0.0309 ± 0.022	0.019 ± 0.023	0.015 ± 0.018	0.017 ± 0.019	0.026 ± 0.030	0.845
TG in extra-large VLDL	0.096 ± 0.0689	0.059 ± 0.071	0.048 ± 0.058	0.0518 ± 0.060	0.083 ± 0.095	0.648
**Extra-extra-large VLDL particle**	$3.321 \times 10^{-10} \pm 2.062 \times 10^{-10}$	$2.033 \times 10^{-10} \pm 2.521 \times 10^{-10}$	$1.607 \times 10^{-10} \pm 1.892 \times 10^{-10}$	$1.783 \times 10^{-10} \pm 1.945 \times 10^{-10}$	$2.665 \times 10^{-10} \pm 2.973 \times 10^{-10}$	0.704
Cholesterol in extra-extra-large VLDL	0.012 ± 0.008	0.007 ± 0.008	0.0058 ± 0.0066	0.007 ± 0.007	0.010 ± 0.011	0.944
TG in extra-extra-large VLDL	0.050 ± 0.031	0.031 ± 0.038	0.024 ± 0.029	0.027 ± 0.029	0.040 ± 0.045	0.677

Quantitative variables are expressed as means ± standard deviations. The *p* value was adjusted by age, sex and BMI.
